# Predictors of Health Care Service Quality among Women Insured Under Ghana’s National Health Insurance Scheme

**DOI:** 10.29024/aogh.2371

**Published:** 2018-11-05

**Authors:** Martin Amogre Ayanore, Richard Ofori-Asenso, Amos Laar

**Affiliations:** 1 Department of Family and Community Health, School of Public Health, University of Health and Allied Sciences, Hohoe, GH; 2 Department of Epidemiology and Preventive Medicine, Monash University, Melbourne, AU; 3 Department of Population Family and Reproductive Health, School of Public Health, College of Health Sciences, University of Ghana, GH; 4 Centre for Health Policy Advocacy, Innovation and Research in Africa (CHPAIR-Africa), Accra, GH

## Abstract

**Background:**

Insured women in Ghana are more likely to use maternity care services than their uninsured counterparts. To improve service quality among insured women in Ghana, better understanding of the factors that predict quality standards of primary health care services is essential.

**Objective:**

To examine predictors of health care service quality among insured women under the National Health Insurance Scheme (NHIS) in Ghana.

**Methods:**

Data from the 2014 Ghana Demographic Health Survey was analysed. Cluster analysis was applied to construct a dependent variable; service care quality. Socio-demographic/background characteristics were used as independent variables. Descriptive and inferential analyses were performed followed by multiple regression to predict service quality among the insured population of women aged 15–49 years. SPSS version 21 was used during the clustering while STATA version 14 was used to perform the inferential and regression analyses.

**Findings:**

A total of 5,457 women with valid health insurance were included in the analysis. Overall, geographical region of respondents was significant to expressions of insured service quality (χ^2^ = 495.4, p ≤ 0.001). Literacy levels were significant at χ^2^ = 69.232 and p < 0.001 for service quality. On place of residence, the estimation showed urban residency to be more positively correlated with indicating quality ratings of health services compared to rural residency (χ^2^ = 70.29, p < 0.001). Highest educational level had the highest predictive influence (coefficient = 0.15) on women’s views about the quality of health care services.

**Conclusions:**

A health insurance system that shifts towards introducing valued-based care models for patients, insurers, and health care providers could be supportive in improving the quality of healthcare delivered to Ghanaians.

## Introduction

In developing countries, women’s perceptions of the quality of health care services is known to be multidimensional [1], and is influenced by a myriad of factors [1,2,3]. To meet demands for patient-centred care services, a fundamental shift from ignoring patients’ perceptions and demands [2] into promoting healthcare environment where the right health professionals are hired and provide the right care is required [4]. Fundamentally, meeting patients’ satisfied demands on health care services drives health care utilization, and subsequently quality ratings on services. Patients’ views and expressions of health care services remain one of the fundamental components of assessing the robustness and ability of health systems to meet growing population health care needs. Studies have suggested that excluding patients’ perspectives in the assessment of health care quality can result in missing valid and unique information about the standards of quality for health care services offered to patients [5]. To promote patient-centred care, health system structures that enable patients to express their values and preferences without censures is vital for quality improvements [6]. The need to incorporate patient-centred perspectives into population- and system-level approaches to advance heath access has been advocated [7]. Barriers and facilitators to patient-centred care in different health contexts are reported [8]. In Ghana, the policy environment on patient-centred care has largely been derived from studies that examined quality and satisfaction attributes for health care services at facility levels [9,10].

Policies on health care quality in Ghana have been largely driven by the desire to achieve a pervasive culture in which the desire to provide quality services is ‘pervasive’ as advocated by the International Society for Quality in Health care [10,11]. The Institutional Care Division (ICD) of the Ghana Health Service (GHS), whose mandate includes the development and implementation of institutional and clinical quality standards, has highlighted several bottlenecks to service quality improvements in Ghana [10]. Studies on patients’ expressions of quality and satisfaction based on clinical outcomes, as well as on patients’ perceptions of previous health care services received have been published [9,12,13,14,15,16,17]. A recent study that assessed health insurance effects on perceived and technical quality of health facilities emphasized the need to actively promote patients’ education as well as comprehensive efforts towards improving health service utilization [18].

Despite the positive effects of Ghana’s National Health Insurance Scheme (NHIS) on maternal and child health outcomes [19,20,21,22,23], there are growing concerns of inequities [24,25], both among the insured and the uninsured populace [26,27]. Although insured women in Ghana are more likely to use maternity care services more than once through the positive mediation of health insurance [22], other studies have reported that some insured population groups may not use health services at all in some settings [28,29,30,31]. To promote insured population groups’ use of health care services, tackling user negative perceptions of health quality and satisfaction preferences are important [31]. Studies on determinants of quality and satisfaction on women’s use of health services in Ghana are reported across general population groups [1,12,15,32]. Other evidence of health insurance impacts on poor households’ access and demands on quality of health care services exists [33,34]. Dixon and colleagues assessed, at a national level, member perceptions of service provision using the 2008 Ghana Demographic Health Survey (GDHS) [35]. A previous study also examined insured and uninsured patients’ satisfaction with the National Health Insurance Scheme (NHIS) in Ghana [14]. However, little is known about insured patients’ expressions of health service quality in Ghana. Thus, this study was conducted to examine the socio-demographic and individual/household background factors that influence health care service quality among insured Ghanaian women.

## Methods

### Data source

Data from the 2014 GDHS was analysed. Detailed survey methodology has been described in the final report by the implementing partners [36]. Women were eligible as respondents if they were aged 15–49 years. DHS surveys apply standardized data collection instruments for data gathering to inform health policy planning and implementation at country levels. The 2014 GDHS applied a two-stage sample design that allowed key national level indicators to be estimated countrywide followed with a systematic sampling of households for enumeration [36]. The two stage sampling yielded 427 clusters (216 in urban areas and 211 in rural areas). An enumeration exercise was conducted between January–March 2014 that selected 30 households from the 427 clusters nationwide, providing a total sample size of 12,831 households. Data collection was conducted between from September to December 2014. The GDHS has undergone modifications since 1988.

In the 2014 survey, the information collected from women included birth history, child mortality, and knowledge and use of family planning and fertility preferences. Others included antenatal, delivery, and postnatal care, breastfeeding and infant feeding practices, vaccinations and childhood illnesses management, women’s empowerment and work outcomes, contraceptive knowledge, awareness and preferences, women and child health and feeding practices, and anthropometric indicators of children. HIV/AIDS knowledge, awareness and practices, children and women medication care, health insurance use, services received with use of health insurance, out-of-pocket (OOP) payments for health, women employment and health outcomes, and socio-demographics of respondents. Our study used data on insured women’s assessment of health services quality and OOP payments for health. Only women who reported having valid health insurance (n = 5,457) were included in the analysis.

### Dependent variable

The dependent variable assessed was insured women’s account of service quality. An index for quality of health care services use was constructed using four variable questions from respondents; pay out-of-pocket for drugs and services, received good service last time, whether service was not covered by the NHIS, and the ease of accessing health care at point of use.

### Construction of dependent cluster membership variables

A two-step cluster procedure for the naturally identifying exploratory natural cases/objects within our data was applied. Cluster analysis is a multivariate statistical technique for grouping cases based on the homogeneity within groups and heterogeneity across groups. The process of clustering enabled the construction of an index the dependent membership variable (perceived health service quality). Cluster analysis has been used previously on understanding focused maternity care in Ghana [22]. Clustering employs an approach to segregate data based on individual responses using log-likelihood distance measure. Since the dataset applied contained both categorical and continuous data, the two-stage clustering procedure is usually preferred [37]. The first clustering approach was the construction of quality index on health services use using the four variable questions described above. The second clustering approach was used to construct the membership variable index for user satisfaction following the same procedures described in the first clustering approach. Overall, common procedure patterns were observed in all estimation. All clustering procedures were set at maximum of four clusters since automatic clustering generated only poor and fair cluster patterns. Four clusters resulted from clustering procedure. Silhouette measure of cohesion and separation for quality showed good quality (> 0.5).

### Independent variables

Individual socio-demographic variables in the analysis included women’s age, marital status, geographical region (10 geographical divisions), religion, highest educational status, religion, parity, literacy, and place of residence. Household variables included in the analysis were decision making for respondent use of contraceptives, person who usually decides how to spend respondents earning, and decision making on meeting respondent health care needs.

### Statistical Analysis

Statistical analysis after clustering procedures was performed in two folds: descriptive and inferential analysis. SPSS version 21 (IBM Corp., Armonk, N.Y., USA) was used during the clustering and exported to STATA version 14 (StataCorp LP, College Station, TX, USA) for further analysis. Further analysis using multiple regression was conducted between service quality significant outcomes to predict service quality among insured. Statistical significant levels were set at (p < 0.05).

### Ethics and data protection issues

Permission for data access and use was granted by the DHS MEASURE program. Ethical consent was obtained from individual respondents during data collection in 2014 by the DHS program implementers. We strictly adhered to the purpose of our request for data access for knowledge dissemination.

## Results

### Respondent’s socio-demographics and cluster findings

Table 1 summarizes the socio-demographic variables assessed in the study. A high proportion of respondents was within the age categories 15–19 and 25–29 years and were married (49.3%). The Upper East Region had the highest record of respondents (11.5%) with the least from the Central Region (7.6%). Secondary level education was high (53%) with an equally higher number of respondents unable to read and write. Urban respondents represented 50.8% while 49.2% were rural residents. The majority of respondents (56.1%) indicated joint decision making with partners in meeting their health care needs. Descriptive cluster groups and socio-demographics are presented in Table 2. A total of 2,673 individual responses were clustered into four cluster groups: cluster 1 (17.62%), 2 (23.08%), 3 (23.5%), and 4 (35.8%). Based on cluster outcomes, the four clusters can be grouped as: adequate care quality for cluster 4, average quality for cluster 3, less adequate for cluster 1, and poor quality for cluster 2 (see Table 2). A high percent (72.8%) of insured women in cluster group 1 indicated they made OOPs for drugs and services. OOP was not reported by those found to have adequate quality (cluster 4). Average care quality cluster (cluster 3) had the highest number of respondents reporting receiving services not covered by the NHIS. Cluster group 2 respondents (14.6%) indicated they received poor quality of care services provided at point of use. Reasons for poor quality ratings in cluster 2 were due to difficulties associated with receiving care (25.6% attributed to this). Age groupings was significant with the quality of health services (χ^2^ = 40.6, p ≤ 0.002).

**Table 1:** Socio-demographic and respondent’s background variables and categories assessed in the study.

Variable	Response	Category	N (%)	Mean	S.D	Variance
	
	Valid (N)				S.E of mean	
	
	Missing					

Age group	Valid (5457)	15–19	973(17.8)	3.595	1.9(0.0161)	3.626
	Missing (0)	20–24	862(15.8)			
		25–29	969(17.77)			
		30–34	817(15.0)			
		35–39	761(14.0)			
		40–44	586(10.73)			
		45–49	489(9.0)			

Marital status	Valid (5457)	Never in union	1663(30.5)	1.067	1.116(0.015)	1.246
	Missing (0)	Married	2689(49.3)			
		Living with partner	649(11.9)			
		Widowed	155(2.8)			
		Divorced	132(2.4)			
		No longer living together/separated	169(3.1)			

Region	Valid (5457)	Western	613(11.2)	5.625	2.838(0.038)	8.053
	Missing (0)	Central	416(7.6)			
		Greater Accra	424(7.8)			
		Volta	544(10.0)			
		Eastern	555(10.2)			
		Ashanti	526(9.6)			
		Brong Ahafo	752(13.8)			
		Northern	517(9.5)			
		Upper East	629(11.5)			
		Upper West	481(8.8)			

Highest educational level		No education	1226(22.5)	1.445	0.911(0.123)	0.831
	Valid (5457)	Primary	945(17.3)			
	Missing (0)	Secondary	2920(53.5)			
		Higher	366(6.7)			

Religion		Christian	4154(76.1)	6.304	0.625(0.008)	0.391
		Islam	1075(19.7)			
		Traditional/spiritualist	98(1.8)			
		No religion	130(2.4)			

Ethnicity	Valid (5457)	Akan	2150(39.4)	21.917	135.135(1.829)	18261.45
	Missing (0)	Ga/Dangme	247(4.5)			
		Ewe	701(12.9)			
		Guan	155(2.8)			
		Mole-Dagbani	1420(26.0)			
		Grusi	294(5.4)			
		Gurma	316(5.8)			
		Mande	71(1.3)			
		Other	103(1.9)			
		Able to read whole sentence	2385(43.7)			
		No card with required language	5(0.1)			
		Blind/visually impaired	3(0.05)			

Type of place of residence	Valid (5457)	Urban	2771(50.8)	1.492	0.5(0.007)	0.25
	Missing (0)	Rural	2686(49.2)			

Parity	Valid (5457)	≤5	5035(92.3)	1.078	0.27(0.004)	0.073
	Missing (0)	6–10	419(7.7)			
		≥11	3(0.05)			

Person who usually decides on respondent’s health care	Valid (3338)	Respondent alone	795(23.8)	2.171	1.025(0.017)	1.051
	Missing (2119)	Respondent and husband/partner	1871(56.1)			
		Husband/partner alone	652(19.5)			
		Someone else	17(0.5)			
		Other	3(0.05)			

**Table 2:** Quality cluster group associations with socio-demographics.

Variable	Quality Cluster outcomes					Pearson Chi Square	

	Cluster 1 Less adequate quality	Cluster 2 Poor quality	Cluster 3 Average quality	Cluster 4 Adequate quality	Total	χ^2^	p-value

	N = 471	N = 617	N = 627	N = 958	N = 2673		

	n(%)	n(%)	n(%)	n(%)	n(%)

**Age in 5-year groups**							

15–19	46(9.8)	54(8.8)	39(6.2)	116(12.1)	255(9.5)	40.6	<0.002
20–24	82(17.4)	105(17.0)	96(15.3)	169(17.6)	452(16.9)		
25–29	106(22.5)	123(19.9)	138(22.0)	203(21.2)	570(21.3)		
30–34	87(18.5)	112(18.2)	110(17.5)	168(17.5)	477(17.8)		
35–39	67(14.2)	90(14.6)	111(17.7)	139(14.5)	407(15.2)		
40–44	43(9.1)	90(14.6)	65(10.4)	80(8.4)	278(10.4)		
45–49	40(8.5)	43(7.0)	68(10.8)	83(8.7)	234(8.8)		
**Region**							

Western	30(6.4)	72(11.7)	63(10.0)	96(10.0)	261(9.8)	495.4	<0.001
Central	28(5.9)	40(6.5)	49(7.8)	79(8.2)	196(7.3)		
Greater Accra	46(9.8)	31(5.0)	84(13.4)	38(4.0)	199(7.4)		
Volta	46(9.8)	118(19.1)	53(8.5)	43(4.5)	260(9.7)		
Eastern	97(20.6)	38(6.2)	49(7.8)	70(7.3)	254(9.5)		
Ashanti	47(10.0)	66(10.7)	67(10.7)	86(9.0)	266(10.0)		
Brong Ahafo	34(7.2)	47(7.6)	140(22.3)	149(15.6)	370(13.8)		
Northern	26(5.5)	111(18.0)	34(5.4)	58(6.1)	229(8.6)		
Upper East	51(10.8)	67(10.9)	37(5.9)	206(21.5)	361(13.5)		
Upper West	66(14.0)	27(4.4)	51(8.1)	133(13.9)	277(10.4)		
**Highest educational level**							

No education	118(25.1)	153(24.8)	99(15.8)	273(28.5)	643(24.1)	78.946	0.001
Primary	70(14.9)	80(13.0)	100(15.9)	201(21.0)	451(16.9)		
Secondary	242(51.4)	334(54.1)	352(56.1)	439(45.8)	1367(51.1)		
Higher	41(8.7)	50(8.1)	76(12.1)	45(4.7)	212(7.9)		
**Ethnicity**							

Akan	178(37.8)	209(33.9)	281(44.8)	360(37.6)	1028(38.5)	150.73	< 0.001
Ga/Dangme	34(7.2)	19(3.1)	28(4.5)	27(2.8)	108(4.0)		
Ewe	60(12.7)	107(17.3)	80(12.8)	79(8.2)	326(12.2)		
Guan	12(2.5)	18(2.9)	30(4.8)	12(1.3)	72(2.7)		
Mole-Dagbani	132(28.0)	159(25.8)	119(19.0)	328(34.2)	738(27.6)		
Grusi	12(2.5)	49(7.9)	52(8.3)	70(7.3)	183(6.8)		
Gurma	23(4.9)	41(6.6)	20(3.2)	38(4.0)	122(4.6)		
Mande	4(0.8)	4(0.6)	3(0.5)	25(2.6)	36(1.3)		
Other	16(3.4)	11(1.8)	14(2.2)	19(2.0)	60(2.2)		
**Literacy**							

Cannot read at all	234(49.7)	278	254(40.5)	554(57.8)	1320(49.4)	69.232	<0.001
Able to read only parts of sentence	33(7.0)	46	56(8.9)	94(9.8)	229(8.6)		
Able to read whole sentence	204(43.3)	293	317(50.6)	309(32.3)	1123(42.0)		
No card with required language	0(0)	0	0(0)	1(0.1)	1(0.04)		
**Place of residence**							

Urban	238(50.5)	343(55.6)	396(63.2)	406(42.4)	1383(51.7)	70.29	<0.001
Rural	233(49.5)	274(44.4)	231(36.8)	552(57.6)	1290(48.3)		

Overall, geographical region of respondents was significant to expressions of insured service quality (χ^2^ = 495.4, p ≤ 0.001). Within the regions, respondents in the Eastern region were more likely to have paid OOP for drugs and services, as they represented a high percent (20.6%) of responses in cluster 1. Poor quality standards were frequently reported in the Volta region (19.1%) as the region was clustered in group 2. The Upper East region had an average quality reports, as more respondents (21.5%) in this cluster are associated with cluster group 3. The Brong-Ahafo region had more respondents (13.5%) clustered in group 4. The analysis further showed that those who could not read at all were mostly involved in clusters 1, 3, and 4, which accounted for 49.7%, 57.8%, and 49.4%, respectively. Those who were able to read whole sentence were also involved in cluster 2 (50.6%). Literacy levels were significant at χ^2^ = 69.232 and p < 0.001. On place of residence, the estimation showed urban residents were more positively correlated with indicating quality ratings of health services compared to rural residents (χ^2^ = 70.29, p < 0.001). Ethnicity and an individual’s educational status had significant associations with the quality of health care services.

## Regression Analysis Findings

Table 3 summarizes multiple regression results to predict insured women’s views on the quality of health services use and socio-demographics. The output shows that the independent variables predict the dependent variable, *F* (10, 2662) = (4.59, p < .05). The highest educational level had the highest predictor influence holding with a coefficient of 0.15; this was followed by place of residence with a coefficient of 0.08.

**Table 3:** Predictors of insured quality of services ratings.

Significant quality of services variables	Coef.	Std. Err.	T	P>|t|	[95% Conf.	Interval]

Age	–0.02498	0.014185	–1.76	0.078	–0.0527962	0.0028348
Marital status	–0.01673	0.022107	–0.76	0.449	–0.0600736	0.0266234
Region	0.026519	0.008075	3.28	0.001	0.0106858	0.042353
Highest education	0.150353	0.100919	1.49	0.136	–0.0475349	0.3482411
Religion	–0.00721	0.03793	–0.19	0.849	–0.0815856	0.0671653
Ethnicity	–0.00017	0.000148	–1.16	0.245	–0.0004632	0.0001184
Educational attainment	–0.08414	0.063149	–1.33	0.183	–0.2079701	0.0396808
Literacy level	–0.08292	0.035395	–2.34	0.019	–0.1523199	–0.01351
Place of residence	0.081462	0.046216	1.76	0.078	–0.0091615	0.1720852
Parity	0.060181	0.085094	0.71	0.479	–0.1066751	0.2270371
Constant value	2.650105	0.284272	9.32	0	2.092688	3.207521

## Discussion

This study examined background and socio-demographic factors that predict health care service quality among women insured under the NHIS in Ghana. Using the 2014 GDHS, we found that quality of health services delivery does influence out-of-pocket payment levels among insured women. Patients’ perceptions of low quality for meeting their health care needs will necessarily equate to patient’s making OOP. This evidence is supported by earlier studies that reported that patients tend to choose services that provide high-quality care [38,39], even when they know they have to endure OOP payments. Insured women who received less adequate care from this study paid more OOP for drugs and services compared to other cluster groups. Expectedly, OOP payments were less reported by those found to have received adequate care quality. Insured women who indicated poor care quality did not frequently report OOP payments. Studies corroborate that patients who pay OOP often receive better or more quicker services than others [40,41]. This has the potential to impact equity issues for health care access in Ghana [42], particularly among insured population groups. We posit that, rising OOP payments among insured population groups to access care may exacerbate drop-out rates and enrolment levels for NHIS in Ghana. Policies that improve NHIS subscription and enrolment and those that ensure continual quality improvements for health services delivered are needed to improve quality care in Ghana. Another addition to existing evidence in Ghana on health insurance and maternal care outcomes from this study is that women’s reports of average or less care quality for primary health care services may be due to their perceived categorizations that some services were not covered under health insurance. The overall effects of poor quality of services among the populace insured under the NHIS in Ghana has the potential to deflate the primary purpose of the policy that is aimed at reducing household catastrophic health effects. A study in Burkina Faso that examined drop-out rates of patients insured under a community-based health insurance scheme found patients’ unsatisfactory quality ratings as one of the causes for drop-outs from the scheme [43]. Providing inclusive structures and opportunities to involve patients in all areas (rural/urban) in knowledge dissemination and operational modalities of the scheme has the potential to address many wrong notions and perceptions of services covered under the NHIS. There is also the need for NHIS managers and policy stakeholders to assess novel ways of bridging inequity issues by not only enrolling pro-poor population groups, but ensuring that services are adequate, timely, and address users’ health needs.

We also found that insured women’s expression of poor quality primary health care services was due to difficulties in accessing care. In examining this further, we can posit that multiple non-service oriented provision and utilization barriers could affect insured client’s quality of services ratings. The potential for health systems in developing countries to be urban and clinic oriented is known [44,45,46]. A more clinic-oriented and urbanized health system has the potential to introduce inequity issues on access among insured groups. In Ghana, a myriad of factors including poor environmental birthing room conditions for women [47], poor provider-patient relations, and clients’ low quality perceptions of clinic level services [48] have been documented to impact general maternal health care utilization. The mechanisms through which the Community Based Health Planning and Services (CHPS) concept in Ghana could be used to reach most rural population needs for improved quality of care requires further evaluation. There is a need for policy shift under the CHPS concept to move beyond access to care to integrating value-based models of care that guarantee quality care improvements for both insured and uninsured populations. The recently launched national quality healthcare strategy geared towards meeting health quality is commendable. Nonethelss, we advocate for further measures aimed at addressing the six major domains of health services quality: safety, efficiency, effectiveness, patient-centred, timeliness, and equity. The domains require multiple stakeholder involvement and a health system that is integrative for it to be able to address unmet needs in Ghana [6,49]. To further address the health needs of both insured and uninsured populations the health system structure (primary, secondary, tertiary) must be adequately aligned with any policy on quality improvements in the medium to long term.

Furthermore, literacy levels among insured women predicted quality of primary health services among the insured from this study. Other studies have established literacy levels and its effects for better health outcomes [50,51]. In other literacy studies, the evidence shows that literacy has the potential to reduce income-related disparities [52,53]. This has potential to positively influence the levels of health care services use. Health care literacy potential for attaining the sustainable development goals (SDGs) in Nepal is documented [54]. By extension, health literacy could be envisaged as a potential catalyst for accelerating progress towards the attainment of universal health coverage (UHC) in developing countries. Socio-demographic factors including geographic region, education, ethnicity, literacy, and place of residence were significant influencers for care quality among the study participants. This is not surprising given the established literature regarding the effects of these factors on health outcomes [55,56,57]. Our results, however, rate educational status as the most significant predictor of care quality among insured Ghanaian women. Educational attainments and its roles in advancing not only health quality needs, but general care access among other groups are also documented [57,58,59].

Overall, an NHIS system that allows for receiving and tracking insured patients’ complaints of the quality of services provided at health facilities will be useful in infusing standards checks with service providers ensuring high standards of care delivery. The system should be able to support the national health insurance authority (NHIA) to institute proper disciplinary measures to poorly rated providers who offer services under the NHIS. A committed and strategic purchasing policy that provides a clear pathway for ensuring allocative efficiency of NHIA funding needs should be formulated and implemented. Our study has some limitations. DHS data collected in 2014 is applied. Our analysis was based on secondary data from DHS, and not all potential variables that could have been of interest were available. Although we established educational attainment as significant predictor to care quality, the inclusion of other contextual socio-demographic variables such as decision making on care seeking, male partners’ support, and control among others may potentially influence the outcome. In addition, our study population included only women of reproductive age and as such our findings may not be applicable to other population groups receiving care under the NHIS.

## Conclusion

The quality of health services influences OOP payment levels among insured women under the NHIS in Ghana. Factors including educational status and literacy level predict high care quality among insured women in Ghana. To address health needs of both insured and uninsured population, health system structures must be adequately aligned with policies on quality improvements in the medium to long term. NHIS managers and policy stakeholders need to assess novel ways of bridging inequity issues among poorly insured groups. A more supportive health insurance system approach that shifts towards introducing value-based care models for patients, insurers, and health care providers could be supportive in improving quality standards. There is a need for further policy discourse and research on best practices on how value-based health financing models could be integrated into Ghana’s health insurance scheme. Such policy shift has potential to improve efficiency and be a critical component of long-term strategies for driving a health for all agenda in Ghana.
